# Public Reporting on the Quality of Care in Patients with Acute Myocardial Infarction: The Korean Experience

**DOI:** 10.3390/ijerph19063169

**Published:** 2022-03-08

**Authors:** Kyunghee Chae, Mira Kim, Byung Ok Kim, Chai Young Jung, Hyun-Jae Kang, Dong-Jin Oh, Dong Woon Jeon, Woo-Young Chung, Cheol Ung Choi, Kyoo-Rok Han, Min-Su Hyon, Hude Quan, Sangmin Lee, Sukil Kim

**Affiliations:** 1 College of Medicine, The Catholic University of Korea, Seoul 06591, Korea; chae9999@gmail.com (K.C.); mkim7111@gmail.com (M.K.); 2 Division of Cardiology, Department of Internal Medicine, Sanggye Paik Hospital, Inje University College of Medicine, Seoul 01757, Korea; byungokim@paik.ac.kr; 3 Biomedical Research Institute, Inha University Hospital, Incheon 22332, Korea; 4 Division of Cardiology, Department of Internal Medicine, Seoul National University Hospital, Seoul 03080, Korea; nowkang@snu.ac.kr; 5 Division of Cardiology, Department of Internal Medicine, Kang Dong Sacred Heart Hospital, Seoul 05355, Korea; ohdjarc@hallym.or.kr (D.-J.O.); krheart@hallym.or.kr (K.-R.H.); 6 Division of Cardiology, National Health Insurance Service Ilsan Hospital, Goyang 10444, Korea; jdw121620@naver.com; 7 Department of Internal Medicine, Seoul National University Boramae Medical Center, Seoul 07061, Korea; chungwy3023@daum.net; 8 Cardiovascular Center, Korea University Guro Hospital, Seoul 08308, Korea; wmagpie@korea.com; 9 Division of Cardiology, Department of Internal Medicine, Soonchunhyang University Seoul Hospital, Seoul 03080, Korea; mshyon@schmc.ac.kr; 10 Department of Community Health Sciences, University of Calgary, Calgary, AB T2N 1N4, Canada; hquan@ucalgary.ca (H.Q.); sarahlee@ucalgary.ca (S.L.)

**Keywords:** public reporting, acute myocardial infarction, quality of care, mortality, ST segment elevation myocardial infarction, NSTEMI

## Abstract

Public reporting is a way to promote quality of healthcare. However, evidence supporting improved quality of care using public reporting in patients with acute myocardial infarction (AMI) is disputed. This study aims to describe the impact of public reporting of AMI care on hospital quality improvement in Korea. Patients with AMI admitted to the emergency room with ICD-10 codes of I21.0 to I21.9 as the primary or secondary diagnosis were identified from the national health insurance claims data (2007–2012). Between 2007 and 2012, 43,240/83,378 (51.9%) patients manifested ST segment elevation myocardial infarction (STEMI). Timely reperfusion rate increased (β = 2.78, *p* = 0.001). The mortality rate of STEMI patients was not changed (β = −0.0098, *p* = 0.384) but that of NSTEMI patients decreased (β = −0.465, *p* = 0.001). Public reporting has a substantial impact on the process indicators of AMI in Korea because of the increased reperfusion rate. However, the outcome indicators such as mortality did not significantly change, suggesting that public reporting did not necessarily improve the quality of care.

## 1. Introduction

Heart disease, including acute myocardial infarction, is the second most common cause of death in Korea [1]. The mortality rate associated with acute myocardial infarction (AMI) in Korea is 8.1%, which is higher than the average mortality rate of 7.5% in the OECD countries [2]. Patients should be treated as soon as possible after the onset of symptoms to reduce the risk of death. Guidelines for AMI diagnosis and treatment have been published by the American College of Cardiology (ACC) and the American Heart Association (AHA) [3] advocate for patients to be treated as soon as possible after the onset of symptoms to reduce the risk of death.

Public reporting has been used to promote the quality of health care worldwide [4,5,6]. The Korean Health Insurance Review and Assessment Service (HIRA) introduced a healthcare performance management program in 2002, with a pilot project of public reporting in 2005 and extended it to private and public hospitals [7]. In Korea, more than 90% of hospitals are private and the percentage of public hospitals is far lower than the OECD average [8]. The main method of payment is fee-for-service (FFS) at both private and public hospitals/clinics, although a prospective payment system (PPS) has been partially introduced.

Studies have shown that public reporting on AMI reduces mortality [9,10] and improves reperfusion rates [11] in patients with AMI. However, other studies have shown no association between public reporting and mortality [11,12,13,14]. Further, studies have demonstrated that public reporting did not improve the quality of process of care [15] and prevented clinicians from treating high-risk patients [14,15,16]. Additionally, the findings of HIRA and the academic society regarding the effect of public reporting in improving the quality of care in patients with AMI are disputed [17].

Further, HIRA introduced the pay-for-performance program in 2011 based on the public reporting scores, which were close to the maximum value with minor variation, suggesting the need to review the appropriateness of public reporting of AMI in improving the quality of care for implementing future programs. To date, the gap between HIRA and the position of healthcare community on the effect of public reporting has yet to be bridged, and therefore the public reporting of AMI has not resumed since its discontinuation in 2012.

Thus, we conducted this study to describe the impact of public reporting on AMI care in improving the hospital quality in Korea. The hypotheses are:

**Hypotheses** **1:** *The public reporting improves the process indicators of AMI.*

**Hypotheses** **2:** *The public reporting reduces the mortality of AMI.*

## 2. Methods

### 2.1. Data Sources

Hospital quality of AMI care data were sourced from HIRA. Data were registered using the computerized system of HIRA for hospital evaluation. Patient demographics, diagnoses, procedures, medication, laboratory results, hospitalization records, and emergency room visits were based on hospital medical data.

### 2.2. Study Population

Patients were included if they were admitted to the emergency room and their primary or secondary diagnosis was assigned International Statistical ICD-10 codes of I21.0 to I21.9. In 2010, the patients with ICD-10 codes in the secondary diagnoses were included. Patients were excluded if they did not have a definitive diagnosis of AMI, were not discharged at the time of data collection, or were diagnosed with AMI after being hospitalized for other disease(s).

Between 2007 to 2012, 21% of patients with definitive AMI, including inpatients and outpatients [18,19,20,21,22,23], met the eligibility criteria in the annual public reporting of AMI care by HIRA, and represented our study population. 99.6% of all tertiary hospitals and 54.5% of all general hospitals in Korea participated in the study [24]. We further stratified our AMI population into patients presenting ischemic symptoms and persistent ST-segment elevation on the electrocardiogram (ECG) for STEMI group and non ST-segment elevation for NSTEMI group according to universal definition of myocardial infarction.

### 2.3. Indicators

The process indicators included rates of reperfusion, timely reperfusion, and oral medication. The reperfusion rates indicate the proportion of patients who underwent reperfusion treatment. Reperfusion rates of patients with STEMI and NSTEMI were calculated, and the correlation between reperfusion rates and mortality in the two groups was analyzed.

Timely reperfusion rate is a treatment performance indicator for STEMI. The treatment of STEMI requires thrombolytic agents and primary percutaneous coronary intervention (P.PCI). Timely reperfusion rate was calculated by combining (1) thrombolytic rate and (2) P.PCI rate. The reference time for reperfusion was shortened in 2010, while the thrombolytic treatment time decreased from 60 min to 30 min and P.PCI time decreased from 120 min to 90 min. The association between mortality of STEMI and timely reperfusion rate was analyzed.

Oral medication rates were defined as the rate of aspirin administered upon arrival and the prescription rate of aspirin and beta blockers at discharge. Oral medications were administered to all of the patients and the association between oral medication rates and mortality was analyzed. Other indicators affecting the care process included the usage rate of ambulance services and the median time from AMI symptoms to door. The median time from arrival at the hospital to thrombolytic treatment and the median time from arrival at the hospital to receiving balloon inflation in the P.PCI procedure for patients with STEMI was computed (Table A1).

The outcome indicators included 30-day mortality rate following admission and 1-year mortality rate from symptom manifestation. Annual mortality rates for six years (2007 to 2012) and monthly mortality rates for four years (2009 to 2012) were calculated.

### 2.4. Statistical Analysis

We investigated the changes in the process and outcome indicators annually or monthly by hospital type (tertiary vs. general) and AMI type (STEMI vs. NSTEMI) for years. Chi-squared analysis and an ANOVA test were used to compare the demographic and clinical characteristics of patients over the years. Linear regression analysis was performed to determine the estimate (*β*) with 95% confidence interval (CI) of the process and outcome indicators by year. A correlation analysis of the association between the process and outcome indicators with respect to hospital type was performed. Multiple regression analyses were performed to determine the association between the process of care and mortality. Only simple linear regression models were created using one of the process indicators due to severe multi-collinearities among the independent variables. The Autoregressive Integrated Moving Average (ARIMA) model (12, 0, 0) was used because of seasonal variation in mortality data. The analysis of NSTEMI was also based on the ARIMA model (0, 0, 1).

SAS (version 9.2, Cary, NC, USA) was used for statistical analysis. The statistical significance was set at *p <* 0.05.

## 3. Results

### 3.1. Characteristics of Patients and Hospitals

Among the total patients analyzed between 2007 and 2012, the least number of patients was evaluated in 2007 because of the inclusion of patients at tertiary hospitals from the last half of the year. The largest number of patients were included in the year 2010 following expansion of the inclusion criteria of targeted subjects with secondary diagnosis of AMI, in addition to the primary diagnosis in the national health insurance claims data. However, the number of eligible patients in 2010 did not significantly increase from the previous year (Table 1).

### 3.2. Process Indicators

**Figure 1:**
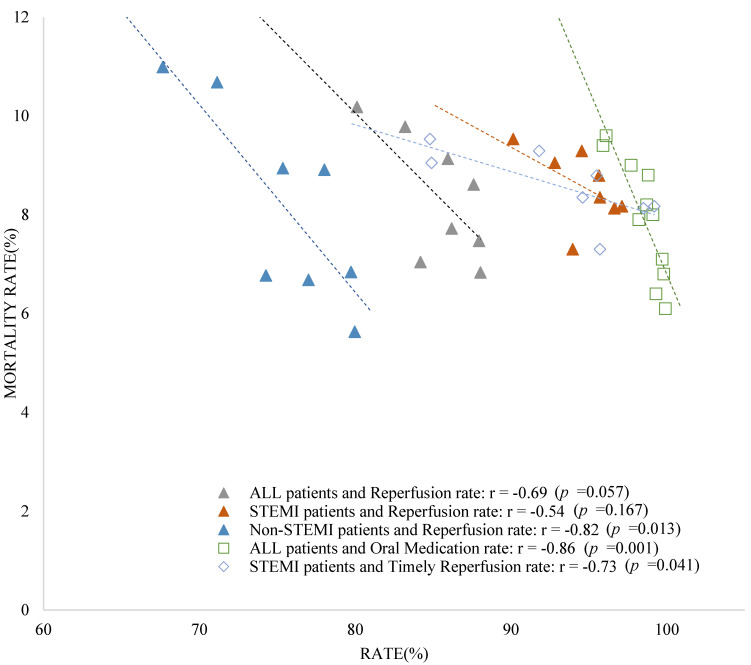
Relationship between annual 30−days mortality and indicators of hospital type. (1) Triangles: Relationship between annual 30−days mortality rate and annual reperfusion rate by hospital type (tertiary and general hospitals) for all patients including STEMI and NSTEMI (2009~2012); (2) Square: Relationship between annual 30−days mortality rate and annual oral medication rate by hospital type (tertiary and general hospitals) for all patients; (3) Rhombus: Relationship between annual 30−days mortality rate in STEMI patients and annual timely reperfusion rate by hospital type (tertiary and general hospitals) (2009~2012).

Of the eligible patients, 84.8% patients underwent reperfusion treatment. Reperfusion treatment was administered to 93.9% STEMI and 75.1% of NSTEMI cases. The reperfusion rate increased to more than 80% during the study period, and was higher in STEMI than in NSTEMI cases (Table 1). Reperfusion rate was increased by 6.6% between 2007 and 2012. Only an increased reperfusion rate of NSTEMI was associated with a decrease in the 30-day mortality of NSTEMI (*p =* 0.013) (Table 2, Figure 1).

The overall timely reperfusion rate showed an increase of 14.0% between 2007 and 2012. The overall timely perfusion rate was 90.9% in 2009 and has exceeded 90.0% since then. In case of tertiary hospitals alone, the timely reperfusion rate increased by 16.3%. In 2009, the rate at the tertiary hospital was 95.7% and steadily increased by 95% since then. The general hospital showed a 13.3% increase in timely reperfusion rate. The increase was associated with a decrease in the 30-day mortality rate of STEMI (*p =* 0.041) (Table 2, Figure 1).

The prescription rates of oral medications increased by 1.4% from 2007 to 2012. The prescription rate of oral medication exceeded 95% since 2007, and no significant difference was found between hospital types. An increase in oral medication rate was associated with a decrease in the 30-day mortality (*p =* 0.001) (Table 2, Figure 1).

The number of patients utilizing an ambulance was 51.5%. The ambulance utilization rate was 51.3% in 2009 and increased by more than 50% since then. The increase was greater in tertiary than in general hospitals. The median AMI symptom-to-door time was the highest at 164 min in 2009 and has since declined. The median symptom-to-door time was longer in tertiary than in general hospitals, with an average difference of 49.7 min per year (*p <* 0.001) (Table 1 and Table 2).

The median door-to-thrombolytic treatment time was longer in general than in tertiary hospitals. The median door-to-P.PCI time was longer in general than in tertiary hospitals (Table 1 and Table 2).

### 3.3. Outcome Indicators

The 30-day mortality rate decreased during the study periods. The 30-day mortality rate in tertiary hospitals did not decrease, whereas it decreased from 2008 to 2012 in general hospitals. It was significantly shorter in tertiary than in general hospitals (*p <* 0.001) (Table 1 and Table 2).

**Figure 2:**
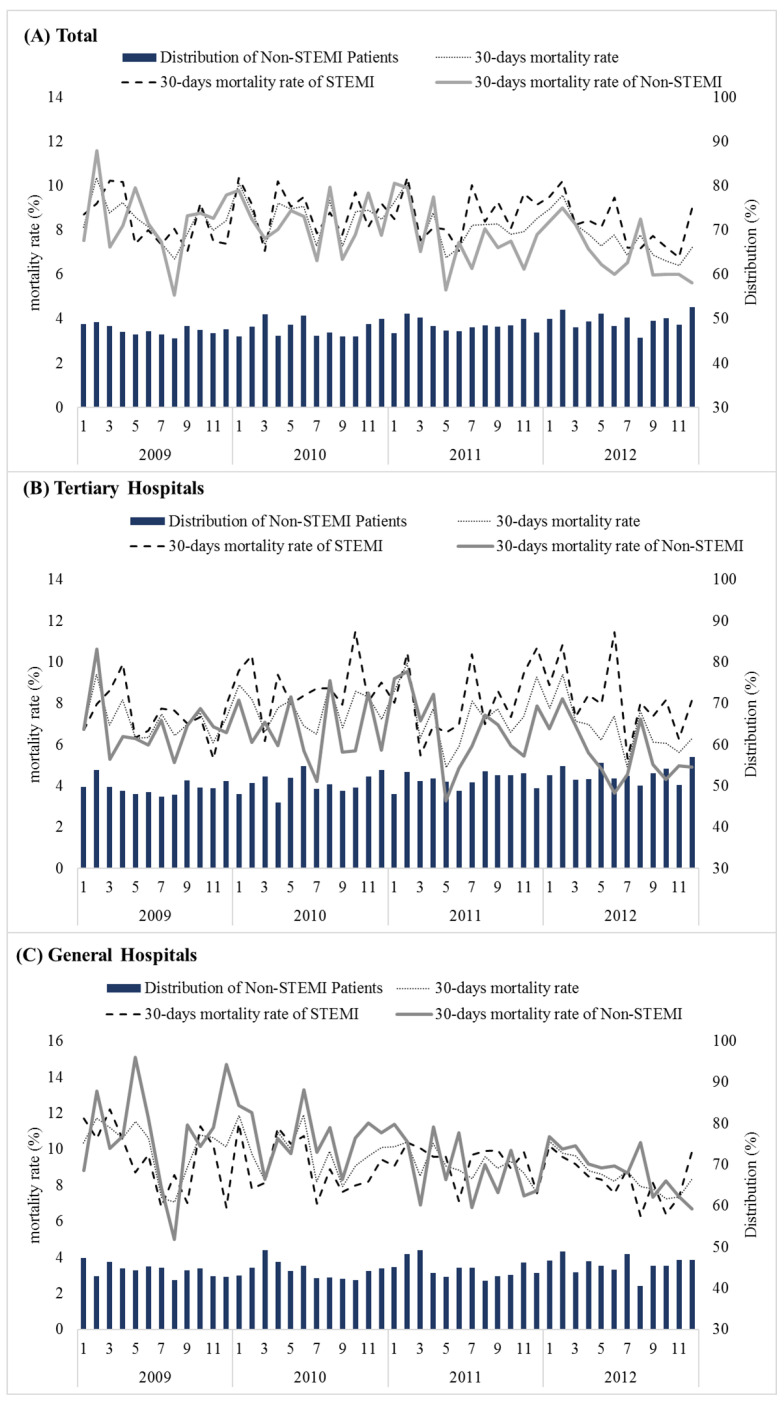
30-day mortality rate and distribution of NSTEMI patients (2009 to 2012). (**A**) Total; (**B**) Tertiary Hospitals; (**C**) General Hospitals.

There were 67,191 patients between 2009 and 2012, including 51.6% of STEMI cases. The overall 30-day mortality rate since admission decreased (*p =* 0.004, ARIMA *p <* 0.001). The 30-day mortality rate for the STEMI did not change (*p =* 0.385, ARIMA *p =* 0.859), but decreased for NSTEMI (*p =* 0.001, ARIMA *p =* 0.001). The 1-year mortality rate decreased during the same time period. The 1-year mortality rate for patients with STEMI did not change, but decreased for NSTEMI cases (Figure 2, Table 3).

## 4. Discussion

### 4.1. Key Findings

Overall, our key findings showed that the public reporting in Korea has a marginal impact on the AMI quality of care improvement. First, the timely reperfusion rate marginally increased during the study period. However, as the reperfusion rate was high (>90%), the margin of increase was limited. Second, the 30-day mortality rate for NSTEMI significantly decreased significantly.

The timely reperfusion rate in 2010 decreased from the previous year presumably due to the 30-min reduction in appropriate treatment time in 2010. The P.PCI rate decreased by 0.5% and the thrombolytic rate decreased by 2%. Thus, the timely reperfusion rate was influenced by a decrease in the thrombolytic rate in the general hospital.

The prescription rate of oral medication increased during the study period. The aspirin prescription at discharge was already greater than 99% in 2007. An increase in the prescription rate of beta blockers might not be regarded as improvement in treatment quality. Despite the controversial side effects of beta-blockers [25], a small number of beta-blockers were administered occasionally even in unnecessary cases to obtain a high score.

### 4.2. Interpretation within the Context of the Wider Literature

During the study period, the 30-day mortality rate significantly decreased among NSTEMI cases but did not significantly change among those with STEMI. There are several possible reasons for the decline in the mortality rate associated with NSTEMI. First, the improved AMI care process resulted in better quality of care. Second, the number of NSTEMI cases who underwent reperfusion increased, which may reflect a greater proportion of admissions involving NSTEMI [26,27,28] or exclusion of NSTEMI cases at the hospitals to increase their performance score. The reduction in mortality rate was higher than the 30-day mortality rate following admission due to STEMI than NSTEMI, or a reduction in the time taken to reach the hospital may have affected the mortality rate. According to Nallamothu’s study, the decreased mortality rate in AMI patients was attributed to changes in population over time [29].

The 30-day mortality rate did not significantly change and the 1-year mortality rate following symptom manifestation decreased. Thus, the long-term benefits of public reporting should be further investigated in larger populations. While the 30-day mortality was higher in STEMI than in NSTEMI cases, the 1-year mortality was higher in NSTEMI than in STEMI. This may potentially be due to the older age of patients with NSTEMI with a greater burden of cardiovascular comorbidities than STEMI [30,31]. Under-utilization of effective cardiac medications and PCI, as well as delays in the time to PCI in patients with NSTEMI may also contribute to this difference in mortality between STEMI and NSTEMI cases [31].

### 4.3. Implications for Policy, Practice and Research

The timely reperfusion rate in tertiary hospitals varied enormously compared with the rate in general hospitals. The mortality rate associated with STEMI decreased in general hospitals due to improved quality of medical care compared with the quality at tertiary hospitals. However, selection bias may have potentially influenced this outcome at the general hospitals as patients reporting favorable treatment outcome may have been selected [14,16]. The tertiary hospitals reported shorter door-to-thrombolytic time, door-to-P.PCI balloon inflation time and better ambulance utilization except symptom-to-door time than the general hospitals. These factors did not lead to a reduction in mortality.

In addition, the data source is another factor determining the outcome. The tertiary hospitals in Korea have an average of 5 or more cardiologists and more than 500 beds [32]. This number is twice the average size than the general clinics. The tertiary setup achieved high rates of timely reperfusion exceeding 95%, in contrast to the general hospitals. The differences in timely reperfusion rate (annual difference of 4−6%) were greater than the differences in the rate of oral medication administered at tertiary and general hospitals, whereas the rate of oral medication was similar at the two hospitals because oral medication is relatively easier to administer than reperfusion treatment. Since timely reperfusion rate requires additional personnel and financial resources, the rate was higher in tertiary hospitals equipped with sufficient medical personnel and resources.

In 2005, ACC/AHA recommended reperfusion treatment for STEMI [3]. The tertiary hospitals immediately implemented the recommendations. The general hospitals with relatively lower levels of reperfusion therapy reported an increase in reperfusion rate during the evaluation period, which reduced mortality. Public reporting leads to improved quality of institutions with scarce medical resources. Public reporting should be used to identify and support hospitals with insufficient resources.

### 4.4. Limitations

Our study has limitations. First, the total number of patients increased in 2010 due to changes in criteria to identify patients with AMI based on secondary diagnosis in addition to primary diagnosis. Since AMI patients identified via secondary diagnosis often failed to receive AMI confirmation at the hospital, only 65% of patients with AMI diagnosis were eligible for this study. However, a majority of AMI patients were selected based on the primary diagnosis. The effects of eligibility criteria, which includes patients with secondary diagnosis after 2010, was insignificant. To ensure the reliability, data were randomly sampled from each hospital and compared with the hospital medical records to establish their accuracy. In case of inconsistency, the error was corrected after careful review. Depending on the indicator, the data for exclusion were determined by an expert advisory council. Second, our study described outcomes of patients with AMI without a comparator group (i.e., outcomes of patients with AMI before public reporting) and thus could not confirm the association between public reporting and quality of care. We included all tertiary hospitals with AMI and most general hospitals offering treatment for AMI in Korea. Almost all of the patients with AMI were included and therefore no hospitals were available for comparison. Since 100% of tertiary hospitals (except only one hospital in 2012) and 50% of general hospitals participated in the public reporting exercise from 2007 to 2012, the findings represent the results of AMI care. Third, due to the lack of patient demographic data, standardized adjusted mortality for case mix index could not be calculated. Thus, there can be various confounders in the relationship between AMI care and outcomes. Fourth, specific factors were not included in our analysis, such as changes in reference time for reperfusion over time and the newly established regional cardio-cerebrovascular center, which might have impacted the AMI care. The government has designated each regional university hospital as a regional cardio-cerebrovascular center to facilitate the rapid treatment of patients with AMI and stroke in all regions. The number of cardio-cerebrovascular centers increased from three in 2008 to 14 in 2018 [33].

## 5. Conclusions

In conclusion, the public reporting system has a substantial impact on the process indicators of AMI in Korea because of the increase in reperfusion rate. However, outcome indicators such as the mortality did not significantly change, suggesting that it did not necessarily improve the quality of care. Disparity exists in quality of care across hospitals, with tertiary care centers equipped with abundant resources offering better quality than general care centers. The variation in public reporting across individual hospitals underscores the need to investigate their management features and practice norms. Further studies are needed on the criteria for treatment and mortality for NSTEMI.

## Figures and Tables

**Table 1:** Characteristics of patients.

Characteristics	Total	Year							Hospital Type		
		2007	2008	2009	2010	2011	2012	*p*	Tertiary Hospitals	General Hospitals	*p*
No. of Source patients	128,327	8633	17,087	19,035	34,321	24,984	24,267		67,646	60,681	
Exclude, No. (%)	44,949 (35.0)	4433 (51.3)	5431 (31.8)	3259 (17.1)	17,866 (52.1)	7722 (30.9)	6238 (25.7)		17,185 (25.4)	27,764 (45.8)	
No. of Eligible patients, No. (%)	83,378 (65.0)	4200 (48.7)	11,656 (68.2)	15,776 (82.9)	16,455 (47.9)	17,262 (69.1)	18,029 (74.3)		50,461 (60.5)	32,917 (39.5)	
Demographic factors											
Age, Mean (SD), year	64.4 (13.2)		64.2 (13.2)	64.5 (13.2)	64.2 (13.2)	64.5 (13.3)	64.3 (13.2)	0.130	64.4 (13.0)	64.3 (13.5)	0.179
Male, No. (%)	55,178 (69.7)		8028 (68.9)	10,806 (68.5)	11,435 (69.5)	12,091 (70.0)	12,818 (71.1)	**<0.001**	32,574 (70.4)	22,845 (69.4)	**0.002**
STEMI ^1^ Group											
STEMI ^1^	43,240 (51.9)	2242 (53.4)	6184 (53.1)	8279 (52.5)	8570 (52.1)	8896 (51.5)	9069 (50.3)	**<0.001**	25,031 (57.9)	18,209 (42.1)	**<0.001**
NSTEMI ^2^	40,138 (48.1)	1958 (46.6)	5472 (46.9)	7497 (47.5)	7885 (47.9)	8366 (48.5)	8960 (49.7)		25,430 (63.4)	14,708 (36.6)	
Hospitals											
Participants hospitals, No. (%)	1005	43	195	211	189	181	186		261 (26.0)	744 (74.0)	
Patients by hospital, Mean (SD)	83 (106.6)	97.7 (61.1)	59.8 (98.8)	74.8 (106.2)	87.1 (105.8)	95.4 (111.3)	96.9 (115.2)	**0.004**	193.3 (130.5)	44.2 (60.1)	**<0.001**
Process											
Reperfusion patients, No. (%)	70,738 (84.8)	3410 (81.2)	9484 (81.4)	13,012 (82.5)	13,967 (84.9)	15,029 (87.1)	15,836 (87.8)	<0.001	43,086 (85.4)	27,652 (84.0)	**<0.001**
STEMI ^1^ patients	40,609 (93.9)	2016 (89.9)	5618 (90.8)	7639 (92.3)	8083 (94.3)	8508 (95.6)	8745 (96.4)	**<0.001**	23,681 (94.6)	16,928 (93.0)	**<0.001**
NSTEMI ^2^ patients	30,129 (75.1)	1394 (71.2)	3866 (70.7)	5373 (71.7)	5884 (74.6)	6521 (77.9)	7091 (79.1)	**<0.001**	19,405 (76.3)	10,724 (72.9)	**<0.001**
Ambulance utilization, No. (%)	35,791 (51.5)	648 (35.8)	3738 (46.6)	7075 (51.3)	7441 (51.5)	8226 (53.6)	8663 (53.8)	**<0.001**	20,917 (52.3)	14,874 (50.3)	**<0.001**
Symptoms to door time, Median (IQR), minute	151 (286.0)	125 (318.0)	156 (298.0)	164 (290.0)	156 (289.0)	150 (278.0)	141 (273.0)	**<0.001**	165 (286.0)	134 (274.0)	**<0.001**
Door to Thrombolytic time, Median (IQR), minute	29 (28.0)	48 (45.0)	42 (35.0)	40 (33.0)	27 (16.0)	27 (7.0)	27 (6.0)	**<0.001**	29 (23.0)	30 (38.0)	0.018
Door to P.PCI balloon inflation time, Median (IQR), minute	69 (36.0)	89 (92.5)	84 (64.0)	77 (48.0)	68 (34.0)	63 (31.0)	61 (30.0)	**<0.001**	68 (36.0)	70(36.0)	0.001
Outcomes											
30-days mortality from admission, No. (%)	5211 (7.7)	139 (7.9)	668 (8.6)	1029 (7.7)	1113 (7.9)	1157 (7.7)	1105 (7.0)	**<0.001**	2698 (6.9)	2522 (8.8)	**<0.001**

Note. Bold values are statistically significant (*p <* 0.05). ^1^ ST-segment elevation on the electrocardiogram; ^2^ Non ST-segment elevation. Of the 128,327, 65% were eligible for the study. The average age was 64 years (SD13.2) and the number of STEMI cases was 51.9%. The number of tertiary patients was 60.5%. An average number of 44 tertiary hospitals and 149 general hospitals participated in the study. The average number of patients was 193 (SD130.5) in each tertiary participating hospital and 45 (SD60.1) in case of general hospital. The average number of patients from the participating tertiary hospital was four-fold greater than the number of patients from the general hospital (Table 1).

**Table 2:** Association between year and process and outcome indicators.

Indicators	Year						Linear Regression by Year		
	2007	2008	2009	2010	2011	2012	Estimate (*β)*	(95% CI)	*p*
Reperfusion rate									
All patients	81.2	81.4	82.5	84.9	87.1	87.8	1.50	(1.01, 1.99)	**0.001**
Tertiary hospitals	81.2	81.9	84.2	86.2	87.9	88	1.55	(1.11, 2.00)	**0.001**
General hospitals		79.9	80.1	83.2	85.9	87.6	2.12	(1.26, 2.98)	**0.004**
Reperfusion rate of STEMI ^1^									
All patients	89.9	90.8	92.3	94.3	95.6	96.4	1.40	(1.16, 1.63)	**<0.001**
Tertiary hospitals	89.9	91.8	94	95.6	96.6	97.1	1.49	(1.05, 1.93)	**0.001**
General hospitals		88.6	90.1	92.8	94.5	95.7	1.85	(1.41, 2.29)	**0.001**
Reperfusion rate of NSTEMI ^2^									
All patients	71.2	70.7	71.7	74.6	77.9	79.1	1.83	(0.96, 2.70)	**0.004**
Tertiary hospitals	71.2	71.5	74.3	77	79.7	80	2.04	(1.42, 2.66)	**0.001**
General hospitals		68.1	67.7	71.1	75.4	78	2.76	(1.29, 4.23)	**0.009**
Timely Reperfusion rate									
All patients	82.9	86.1	90.9	90.6	95.3	96.9	2.78	(1.97, 3.59)	**0.001**
Tertiary hospitals	82.9	88.7	95.7	95.5	98.5	99.2	3.16	(1.45, 4.48)	**0.011**
General hospitals		81.3	84.8	84.9	91.8	94.6	3.36	(1.67, 5.05)	**0.008**
Thrombolytic rate within 30 min									
All patients	70.3	79.7	81.9	79.9	88.4	90	3.50	(1.50, 5.50)	**0.008**
Tertiary hospitals	70.3	86.4	91.2	93.3	97.3	96.6	3.92	(1.67, 6.17)	**0.008**
General hospitals		69	71.5	61.9	79.6	84.2	3.85	(−3.54,11.24)	0.196
P.PCI ^3^ rate within 90 min									
All patients	85.3	86.9	91.7	91.2	95.7	97.3	2.45	(1.68, 3.23)	**0.001**
Tertiary hospitals	85.3	88.9	96	95.7	98.6	99.3	2.82	(1.4, 4.24)	**0.005**
General hospitals		83	86	86.1	92.6	95.2	3.1	(1.47, 4.72)	**0.009**
Oral Medication rate									
All patients	98.2	97.9	98	98.8	99.4	99.6	0.35	(0.11, 0.59)	**0.015**
Tertiary hospitals	98.2	98.7	99.3	99.7	99.8	99.9	0.35	(0.19, 0.50)	**0.003**
General hospitals		95.9	96.1	97.7	98.8	99.1	0.91	(0.49, 1.33)	**0.006**
Aspirin at arrival rate									
All patients	98	98.1	98.6	99.1	99.6	99.4	0.34	(0.19, 0.49)	**0.003**
Tertiary hospitals	98	98.8	99.7	99.8	99.9	100	0.38	(0.12, 0.64)	**0.015**
General hospitals		96.6	97.2	98.3	99.2	98.9	0.66	(0.22, 1.10)	**0.018**
Aspirin prescribed at discharge									
All patients	99.5	99.4	99.3	99.6	99.6	99.8	0.07	(−0.02, 0.16)	0.098
Tertiary hospitals	99.5	99.6	99.6	99.9	99.8	99.9	0.08	(0.03, 0.14)	**0.014**
General hospitals		98.9	98.9	99.1	99.3	99.6	0.18	(0.08, 0.28)	**0.010**
Beta-blocker prescribed at discharge									
All patients	96.1	96	95.7	97.7	98.8	99.5	0.78	(0.29, 1.28)	**0.012**
Tertiary hospitals	96.1	97.7	98.7	99.3	99.6	99.9	0.72	(0.38, 1.07)	**0.004**
General hospitals		91.1	91.3	95.4	97.8	98.9	2.21	(1.18, 3.25)	**0.007**
30-days mortality rate from admission									
All patients	7.9	8.6	7.7	7.9	7.7	7.0	−0.20	(−0.46, 0.06)	0.101
Tertiary hospitals	7.9	8.2	6.4	7.1	6.8	6.1	−0.36	(−0.72, 0.01)	0.050
General hospitals		9.4	9.6	9.0	8.8	8.0	−0.36	(−0.65, −0.07)	**0.030**
Ambulance utilization rate									
All patients	35.8	46.6	51.3	51.5	53.6	53.8			
Tertiary hospitals	35.8	46.8	52.4	52.5	55.9	55.7			
General hospitals		46	50	50.2	50.9	51.6			
Symptoms to door Median time (minute)									
All patients	125	156	164	156	148	140			
Tertiary hospitals	125	163	176	173	162	158			
General hospitals		139	145	135	131	121			
Door to Thrombolytic Median Time (minute)									
All patients	49.5	46	40	27	27	27			
Tertiary hospitals	50	38	33	26	26	27			
General hospitals		54	48	29	27	26			
Door to P.PCI balloon inflation Median Time (minute)									
All patients	106.5	85	76	67	62	61			
Tertiary hospitals	107	82	72	65	60	60			
General hospitals		88	81	70	65	63			

Note. Bold values are statistically significant (*p <* 0.05). ^1^ ST-segment elevation on the electrocardiogram; ^2^ Non ST-segment elevation; ^3^ Primary percutaneous coronary intervention.

**Table 3:** Linear regression analysis of factors associated with mortality rate (2009 to 2012).

Type	No		Regession for Observation by Time (Year Month)			Regession for ARIMA ^1^ Model by Time (Year Month)		
		No. of Cases	Estimate (β)	(95% CI)	*p* Value	Estimate (β)	(95% CI)	*p* Value
30-day mortality rate from admission								
Total								
All	67,191	5511	−0.00093	(−0.00155, −0.00031)	**0.004**	−0.00140	(−0.00187, −0.00093)	**<0.001**
STEMI ^2^	34,653	2966	−0.00032	(−0.00105, 0.00041)	0.385	−0.00005	(−0.00064, 0.00053)	0.859
NSTEMI ^3^	32,538	2545	−0.00152	(−0.00242, −0.00062)	**0.001**	−0.00136	(−0.00216, −0.00057)	**0.001**
Distribution of NSTEMI ^3^ patients	67,191	32,538	0.00176	(0.00065, 0.00288)	**0.003**	0.00145	(0.00067, 0.00224)	**0.001**
Tertiary Hospitals								
All	37,595	2742	−0.00051	(−0.00129, 0.00028)	0.202	0.00000	(−0.00062, 0.00062)	0.999
STEMI ^2^	18,378	1493	0.00035	(−0.00071, 0.00141)	0.508	0.00075	(−0.00003, 0.00153)	0.061
NSTEMI ^3^	19,247	1249	−0.00118	(−0.00223, −0.00013)	**0.029**	−0.00075	(−0.00159, 0.00009)	0.081
Distribution of NSTEMI ^3^ patients	37,595	19,247	0.00288	(0.00149, 0.00427)	**<0.001**	0.00263	(0.00173, 0.00353)	**<0.001**
General Hospitals								
All	29,596	2769	−0.00164	(−0.00240, −0.00088)	<0.001	−0.00158	(−0.00229, −0.00088)	<0.001
STEMI ^2^	16,305	1473	−0.00119	(−0.00216, −0.00021)	**0.018**	−0.00105	(−0.00189, −0.00020)	**0.017**
NSTEMI ^3^	13,291	1296	−0.00221	(−0.00351, −0.00091)	**0.001**	−0.00218	(−0.00343, −0.00094)	**0.001**
Distribution of NSTEMI ^3^ patients	29,596	13,291	0.00077	(−0.00065, 0.00219)	0.286	0.00046	(−0.00069, 0.00161)	0.427
1-year mortality rate from Symptoms								
Total								
All	67,191	9396	−0.00002	(−0.00003, −0.00001)	**0.002**	−0.00001	(−0.00002, 0.00000)	**0.007**
STEMI ^2^	34,653	4379	−0.00001	(−0.00002, 0.00000)	0.073	−0.00001	(−0.00002, 0.00000)	0.237
NSTEMI ^3^	32,538	5017	−0.00003	(−0.00004, −0.00001)	**<0.001**	−0.00002	(−0.00003, −0.00001)	**0.001**
Tertiary Hospitals								
All	37,595	4938	−0.00001	(−0.00002, 0.00000)	**0.046**	−0.00001	(−0.00002, 0.00000)	0.243
STEMI ^2^	18,378	2253	0.00000	(−0.00002, 0.00001)	0.520	0.00000	(−0.00001, 0.00001)	0.903
NSTEMI ^3^	19,247	2685	−0.00002	(−0.00003, 0.00000)	**0.010**	−0.00001	(−0.00002, 0.00000)	0.061
General Hospitals								
All	29,596	4458	−0.00003	(−0.00004, −0.00001)	**<0.001**	−0.00002	(−0.00004, −0.00001)	**<0.001**
STEMI ^2^	16,305	2126	−0.00002	(−0.00003, 0.00000)	**0.016**	−0.00001	(−0.00003, 0.00000)	**0.034**
NSTEMI ^3^	13,291	2332	−0.00004	(−0.00006, −0.00002)	**<0.001**	−0.00004	(−0.00005, −0.00002)	**<0.001**

Note. Bold values are statistically significant (*p <* 0.05). ^1^ Autoregressive Integrated Moving Average (ARIMA) model; ^2^ ST-segment elevation on the electrocardiogram; ^3^ Non ST-segment elevation. The 30-day mortality rate of STEMI did not significantly change in tertiary hospitals (*p =* 0.508, ARIMA *p =* 0.061), although it was decreased for NSTEMI cases significantly based on observational data (*p =* 0.029). However, the ARIMA model was not significant (*p =* 0.081). In case of general hospitals, the 30-day mortality rate declined for both STEMI (*p =* 0.018, ARIMA *p =* 0.017) and NSTEMI (*p =* 0.001, ARIMA *p =* 0.001). The total number of NSTEMI cases increased (*p =* 0.003, ARIMA *p <* 0.001). In tertiary hospitals, the number of NSTEMI cases increased (*p <* 0.001, ARIMA *p <* 0.001), but did not change in the general hospitals (*p =* 0.281, ARIMA *p =* 0.427) (Figure 2, Table 3).

## Data Availability

No new data were generated or analyzed in support of this review.

## Appendix A

**Table A1:** Study variables.

Variables	Numerator	Denominator (Case of Population)
Population		
Source population	No. of patients identified from claim data	
Eligible patients	No. of patients diagnosed with AMI	
Exclude	No. of excluded patients	
Male patients	No. of male patients	
Age	Mean, SD of patients	
Age of males	Mean, SD of male patients	
Age of females	Mean, SD of female patients	
STEMI ^1^ patients	ST elevation on ECG or new onset LBBB	
Patients treated with reperfusion	No. of patients exposed to reperfusion	
Reperfusion of STEMI patients	No. of STEMI patients treated with reperfusion	
Reperfusion of NSTEMI ^2^ patients	No. of NSTEMI patients treated with reperfusion	
Monthly patients	Monthly number of patients	
Monthly number of STEMI patients	Monthly number of STEMI patients	
Monthly number of non-STEMI patients	Monthly number of NSTEMI patients	
Process		
Timely reperfusion rate (A = A1 + A2)		STEMI
Thrombolytic rate within 30 min (A1)	within 30 min of arrival (from 2010 to 2012)within 60 min of arrival (from 2007 to 2009)	Thrombolytic therapy within 6 h of hospital arrival of STEMI
P.PCI rate within 90 min (A2)	within 90 min of arrival (from2010 to 2012)within 120 min of arrival (from 2007 to 2009)	P.PCI within 12 h of hospital arrival of STEMI
Oral medication (B = B1 + B2 + B3)		Eligible
Aspirin at arrival (B1)	Aspirin administered within 24 h of hospital arrival	
Aspirin prescribed at discharge (B2)	prescribed at discharge	
Beta-blocker prescribed at discharge (B3)	prescribed at discharge	
Ambulance utilization rate	No. of patients hospitalized by ambulance	Eligible
Symptom-to-door (S2D) median time (minutes)	Symptom-to-door median time (minutes)	Eligible
Door-to-thrombolytic(D2T) median time (minutes)	Door to thrombolytic (D2T) median time (minutes)	STEMI
Door-to-P.PCI balloon inflation (D2B) median time (minutes)	Door-to-P.PCI balloon inflation (D2B) median time (minutes)	STEMI
Outcomes		
30-day mortality rate following admission	No. of patients who died within 30 days of hospitalization	Eligible
In-hospital mortality rate	No. of patients who died during hospitalization	
Monthly 30-days mortality rate from admission	Number of patients dying within 30 days of hospitalization	Eligible
1-year mortality following symptoms	Number of patients dying within 1 year of symptom manifestation	

^1^ ST-segment elevation on the electrocardiogram; ^2^ Non ST-segment elevation.
